# Physiological and genomic evidence of cysteine degradation and aerobic hydrogen sulfide production in freshwater bacteria

**DOI:** 10.1128/msystems.00201-23

**Published:** 2023-06-07

**Authors:** Patricia Q. Tran, Samantha C. Bachand, Jacob C. Hotvedt, Kristopher Kieft, Elizabeth A. McDaniel, Katherine D. McMahon, Karthik Anantharaman

**Affiliations:** 1 Department of Bacteriology, University of Wisconsin-Madison, Madison, Wisconsin, USA; 2 Freshwater and Marine Sciences Doctoral Program, University of Wisconsin-Madison, Madison, Wisconsin, USA; 3 Microbiology Doctoral Training Program, University of Wisconsin-Madison, Madison, Wisconsin, USA; 4 Department of Civil and Environmental Engineering, University of Wisconsin-Madison, Madison, Wisconsin, USA; E O Lawrence Berkeley National Laboratory, Berkeley, California, USA

**Keywords:** sulfur, microbial ecology, biogeochemistry, freshwater, cysteine, organosulfur metabolism

## Abstract

The sulfur-containing amino acid cysteine is abundant in the environment, including in freshwater lakes. Biological cysteine degradation can result in hydrogen sulfide (H_2_S), a toxic and ecologically relevant compound that is a central player in biogeochemical cycling in aquatic environments. Here, we investigated the ecological significance of cysteine in oxic freshwater, using isolated cultures, controlled experiments, and multiomics. We screened bacterial isolates enriched from natural lake water for their ability to produce H_2_S when provided cysteine. We identified 29 isolates (Bacteroidota, Proteobacteria, and Actinobacteria) that produced H_2_S. To understand the genomic and genetic basis for cysteine degradation and H_2_S production, we further characterized three isolates using whole-genome sequencing (using a combination of short-read and long-read sequencing) and tracked cysteine and H_2_S levels over their growth ranges: *Stenotrophomonas maltophilia* (Gammaproteobacteria), *S. bentonitica* (Gammaproteobacteria), and *Chryseobacterium piscium* (Bacteroidota). Cysteine decreased and H_2_S increased, and all three genomes had genes involved in cysteine degradation. Finally, to assess the presence of these organisms and genes in the environment, we surveyed a 5-year time series of metagenomic data from the same isolation source (Lake Mendota, Madison, WI, USA) and identified their presence throughout the time series. Overall, our study shows that diverse isolated bacterial strains can use cysteine and produce H_2_S under oxic conditions, and we show evidence using metagenomic data that this process may occur more broadly in natural freshwater lakes. Future considerations of sulfur cycling and biogeochemistry in oxic environments should account for H_2_S production from the degradation of organosulfur compounds.

**IMPORTANCE:**

Hydrogen sulfide (H_2_S), a naturally occurring gas with both biological and abiotic origins, can be toxic to living organisms. In aquatic environments, H_2_S production typically originates from anoxic (lacking oxygen) environments, such as sediments, or the bottom layers of thermally stratified lakes. However, the degradation of sulfur-containing amino acids such as cysteine, which all cells and life forms rely on, can be a source of ammonia and H_2_S in the environment. Unlike other approaches for biological H_2_S production such as dissimilatory sulfate reduction, cysteine degradation can occur in the presence of oxygen. Yet, little is known about how cysteine degradation influences sulfur availability and cycling in freshwater lakes. In our study, we identified diverse bacteria from a freshwater lake that can produce H_2_S in the presence of O_2_. Our study highlights the ecological importance of oxic H_2_S production in natural ecosystems and necessitates a change in our outlook on sulfur biogeochemistry.

## INTRODUCTION

In most natural environments, hydrogen sulfide gas (H_2_S) production is usually attributed to defined groups of bacteria and archaea (1, 2) and occurs primarily in anoxic environments. During the process of dissimilatory sulfate reduction, sulfate acts as a terminal electron acceptor and is converted to hydrogen sulfide. However, other pathways for H_2_S production exist, namely assimilatory sulfate reduction, in which H_2_S contributes to cell growth and increased biomass, and the desulfurylation (desulfurization) of sulfur-containing amino acids, such as cysteine, which can lead to production of pyruvate, ammonia, and H_2_S (3). It is believed that assimilatory sulfate reduction contributes to growth but does not release H_2_S from the cell, while dissimilatory sulfate reduction and cysteine degradation can both contribute to growth and release of ecologically relevant nitrogen and sulfur compounds into the ecosystem.

Microbes are responsible for several steps of the assimilatory and dissimilatory pathways, which enable sulfur intermediates (sulfate, thiosulfate, and hydrogen sulfide) to flow in the environment. Sulfur cycling in freshwater ecosystems can be ecologically significant, especially in places where strong redox gradients exist (4). For example, in high arctic lakes, intermediate sulfur compounds (other than sulfate and hydrogen sulfide) are suggested to serve as biogeochemical hubs, because more organisms had genes to transform the intermediates, than for sulfate reduction and H_2_S oxidation (5). Cysteine, a sulfur-containing amino acid, is proposed to be an overlooked source of both carbon (6) and sulfur. Additionally, seston (particles in water comprised both living and non-living organisms) contain organosulfur-containing lipids, which settle into the sediments, and contributes to the sulfur pool, even in highly oligotrophic lakes such as Lake Superior (7). We also note that some of these transformations can occur abiotically as well. For example, measuring sulfur isotopes can reveal if a compound has been formed through biological or abiotic processes, and abiotic transformations of sulfur compounds have been observed in geothermal spring environments (8). Therefore, the sulfur cycle can be complex to parse out because it is driven by both biotic and abiotic factors and has multiple intermediates, for which the details of multiple specific pathways remain to be studied.

In seasonally stratified lakes consisting of oxygenated warm water (epilimnion) floating atop colder anoxic waters (hypolimnion), H_2_S is often abundant in the hypolimnion (9, 10) due to oxygen demand driving terminal electron acceptor depletion. However, an overlooked player in the pool of available H_2_S is the use of organosulfur compounds, such as cysteine, by microbes. Cysteine is required to produce many proteins and is also important for protein structure. It is one of the two amino acids (methionine being the other) that contains a sulfur group; however, the sulfhydryl group on cysteine is more reactive and can lead to H_2_S formation. Methionine can also lead to H_2_S formation, for example, in the human body (11, 12).

Like all amino acids, cysteine also contains an amine group that will form ammonia once the molecule is degraded. As such, cysteine degradation (desulfurylation) by microbes leads to H_2_S production. H_2_S is ecologically relevant because it can be toxic to plants and animals. During periods of anoxia, H_2_S can accumulate to levels beyond the threshold for living organisms and can cause massive fish kills (13). Unlike other H_2_S sources, cysteine desulfurylation can occur under oxic conditions (such as in *Escherichia coli*) (14), thereby expanding the environmental scope of this sulfur pool. Indeed, cysteine can be desulfurylated under oxic conditions in the laboratory, but the natural prevalence of this process in freshwater lakes and other oxic environments remains unknown. We expect that H_2_S production in oxic environments (during the mixed water column periods of the year and throughout the stratified period in the mixed epilimnion) could result from cysteine breakdown by microbes.

**Fig 1:**
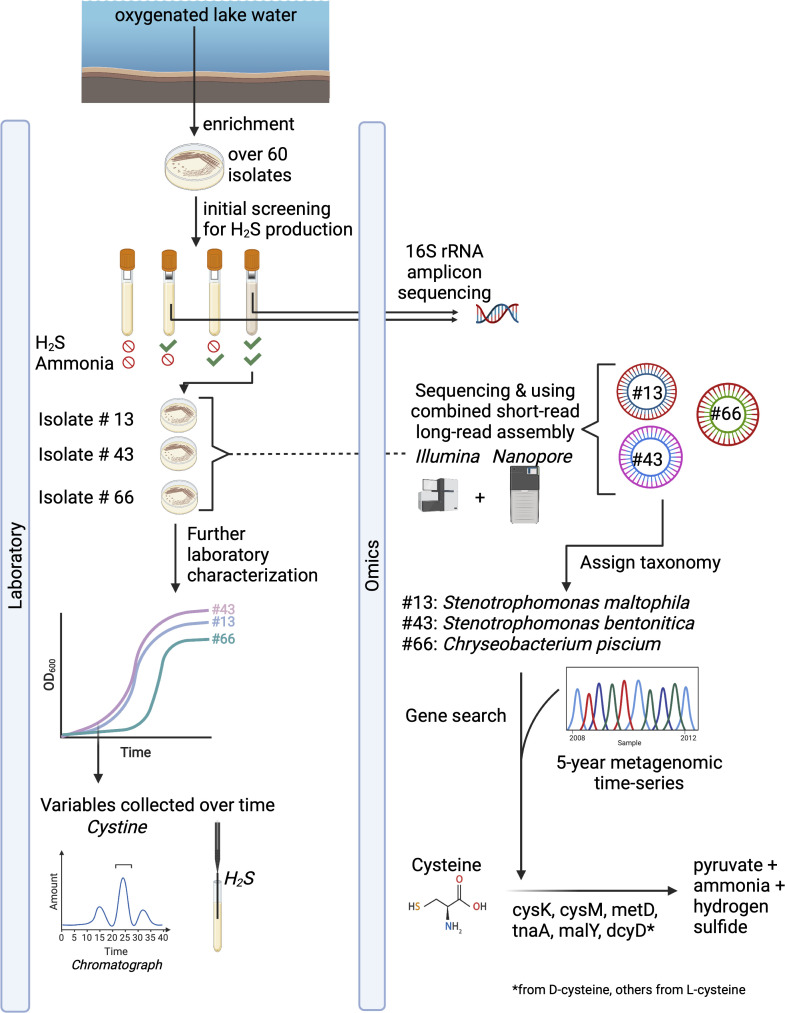
Overview of physiological and genomic methods to characterize hydrogen sulfide production from isolates from freshwater lakes. Isolates were enriched from oxygenated lake water, resulting in about over 60 isolates. Isolates were screened for H_2_S and ammonia production using qualitative H_2_S and ammonia production assays. Isolates that produced H_2_S were selected for 16S rRNA gene sequencing. Then, based on the taxonomic results from 16S rRNA gene sequencing, three distinct isolates were selected for whole-genome sequencing using a combination of short- and long-read sequencing. Genome characterization of functional potential and taxonomic classification was conducted on the isolates. Screening of genes involved in cysteine degradation (Table S4 at https://doi.org/10.6084/m9.figshare.21711491) was conducted in the isolates and a 5-year metagenomic time series of Lake Mendota (2008-2012).

In this study, we investigated the prevalence of organosulfur degradation (desulfurylation) in a freshwater lake, using both laboratory and genomic evidence, to advance our understanding of oxic sulfur cycling in aquatic ecosystems (Fig. 1). First, we grew bacterial isolates enriched from Lake Mendota’s oxic epilimnion to quantify H_2_S and ammonia production, which informs the potential for organosulfur degradation in an oxygenated aquatic environment. We found 18 isolates producing H_2_S under oxic conditions. We selected three H_2_S-producing isolates for detailed characterization using full-genome sequencing and chemical analyses to track cysteine concentrations and H_2_S accumulation during their growth: *Stenotrophomonas maltophilia* (Gammaproteobacteria), *S. bentonitica* (Gammaproteobacteria), and *Chryseobacterium piscium* (Bacteroidota). In all three isolates, cysteine decreased and H_2_S increased over their exponential growth curve under oxic conditions. Finally, we contextualized our laboratory results using a time series of metagenomic data from the same isolation source (Lake Mendota, Madison, WI, USA) to study the temporal importance of organosulfur degradation. We found that genes for cysteine desulfurylation were present and abundant throughout the time series, suggesting that the ability to degrade cysteine is well represented in Lake Mendota.

## MATERIALS AND METHODS

### Enrichment cultures of isolates from a temperate freshwater lake

Lake Mendota (43°06′24″ N 89°25′29″ W) is a temperate eutrophic lake in South Central Wisconsin, in Madison, WI, USA. Lake Mendota is part of the North Temperate Lakes Long-Term Ecological Research Network (https://lter.limnology.wisc.edu/about/lakes). Lake Mendota encounters annual stratification and annual seasonal anoxia in the hypolimnion. Lake water was collected on 14 September 2018 from an integrated water sample (0–12 m) from Lake Mendota at the “Deep Hole” station (43°05′54″, 89°24′'28″), where the maximum depth is 23.5 m. The water samples were collected during stratification from the oxygenated epilimnion. The lake water was collected in preacid washed 2-L sampling bottles using a flexible PVC tube and brought back on shore within hours for immediate processing. Serial dilution was performed, and lake bacteria were grown on PCB (plate count media broth) agar media at room temperature (~21°C), in the lab under the light. The PCB media was made of: 1 L water, 5 g/L of yeast extract, 10 g/L of tryptone, and 2 g/L of dextrose/D-glucose. If grown on solid media, 10 g of agar per 1 L media was added. Enrichment resulted in about 60 isolates.

### Screening for cysteine degradation into H_2_S and ammonia

Isolates were able to grow on PCB and Reasoner's 2A agar (R2A media). R2A media is a culture medium for bacteria that typically grow in freshwater. It is less “nutrient-rich” than PCB media and, therefore, slightly closer to natural lake water than PCB. For the screening of the isolates for H_2_S production, we grew them on R2A media. Each isolate had two treatments: grown in R2A media without cysteine for the control and grown in R2A media with amended cysteine as the treatment.

R2A media consisted of (1 L of water) 0.5 g of casein, 0.5 g of dextrose, 0.5 g of starch, 0.5 g of yeast extract, 0.3 g of K_2_HPO_4_, 0.3 g of sodium pyruvate, 0.25 g of peptone, 0.25 g of beef extract, 0.024 g of MgSO_4_, and autoclave. To make the same media for plates, we added 15 g of agar before autoclaving. For controls, isolates were grown in the media without cysteine amendments. For “treatments,” 2 mM cysteine was added.

To assess the amount of cysteine degradation into H_2_S and ammonia, we screened each of the 60 isolates for H_2_S and/or ammonia accumulation. To test H_2_S production, we grew the strains individually in liquid media, in culture tubes with loose fitting caps, thus letting oxygen in. They were also shaken at room temperature. We used lead acetate test strips (Fisher Scientific, Waltham, Massachusetts, USA) to qualitatively assess H_2_S accumulation in the headspace. A darkening of the strip shows that H_2_S was produced. To test ammonia concentrations after 24 h, we measured samples at time 0 and 24 h using Ammonia Salicylate Reagent Powder Pillows and Ammonia Cyanurate Reagent Powder Pillows (Hatch Reagents) and used spectrophotometry at the 655-nm wavelength.

The three isolates were tested by thioglycolate broth test and were determined to be obligate aerobes.

### Identification of H_2_S-producing bacteria using 16S rRNA gene sequencing

Colony PCR and DNA extractions were conducted using the EtNa Crude DNA Extraction and ExoSAP-IT PCR Product Cleanup protocols on the isolates that tested positive for producing H_2_S (10). Full-length 16S rRNA gene products were generated for sequencing using universal 16S rRNA primers (27f, 1492r) (15). DNA concentration yields were measured using the QuBit dsDNA HS assay kit (QuBit). DNA was sequenced at the University of Wisconsin-Madison Biotechnology Center (Madison, WI, USA). The program 4Peaks (16) was used to clean the base pairs by quality checking followed by homology search using BLASTn against the NCBI Genbank database (accessed December 2019) (17) to identify the sequences.

### Detailed characterization of three H_2_S-producing isolates

We selected three isolates that could aerobically produce H_2_S for further detailed characterization. We selected these isolates because some of the 18 isolates that produced H_2_S when grown with cysteine had identical 16S rRNA sequences; therefore, we chose isolates that had distinct 16S rRNA sequences for full-genome sequencing. Additionally, using 16S rRNA gene sequencing of the isolates, only one was assigned to *Stenotrophomonas* sp., and we believed that whole-genome sequencing would enable us to get a higher taxonomic confirmation and more complete information.

We performed DNA extraction using the PowerSoil Powerlyzer kit (Qiagen) without protocol modifications and sent the genomic DNA for whole-genome sequencing at the Microbial Genome Sequencing Center (MIGS; Pittsburg, PA, USA) with combined short-read Illumina and long-read nanopore sequencing. The data were processed by MIGS to assemble the short reads (Illumina Next Seq 2000) and long reads (Oxford Nanopore Technologies [ONT]) into full genomes. Quality control and adapter trimming were performed with Bcl2fastq (Illumina) and Porechop (https://github.com/rrwick/Porechop) for Illumina and ONT MinION sequencing, respectively. Hybrid assembly with Illumina and ONT reads was performed with Unicycler (18). Genome annotation of the three isolates was done with Prokka v.1.14.5 (19), using the --rfam setting.

Genome completeness and contamination were estimated using CheckM v.1.1.3 (20) *lineage_wf*. Taxonomic classification was conducted using GTDB-tk v.0.3.2 (21) with the database release r95. The full-genome taxonomic classification agreed with the prior 16S rRNA gene sequencing results, but we were further able to identify Isolate 43 as *S. bentonitica*. We ran METABOLIC-G v.4.0 (22) to identify genes associated with cysteine degradation and other metabolic pathways.

Growth measurements of the three isolates were measured using OD_600_ with a spectrophotometer, with measurements every 1 h. The isolates were grown in R2A broth media, shaken in an incubator at 27°C. Aliquots were collected over the growth range for cysteine measurements across the growth curves, as described below. A H_2_S microsensor (Unisense) was used to measure H_2_S over time.

### Methods to measure cysteine

Cysteine concentrations were measured as cystine, as described by Hotvedt et al. (23) (https://osf.io/9k8a6/). One of the reasons for measuring cystine instead of cysteine is that in oxic environments, cysteine is oxidized rapidly into cystine (24, 25). Additionally, unless liquid chromatography-mass spectrometry is used, cysteine can be difficult to measure directly. Samples were diluted 5:4:1 Sample:DI H_2_O:DMSO and left at room temperature for at least 24 h. Chromatographic analysis was performed on an Agilent 1260 Infinity II with an Agilent Zorbax Eclipse Plus C18 RRHT 4.6 × 50 mm, 1.8 µm, with Guard column. Column temperature was maintained at 40°C using an Agilent 1260 TCC (G1316A).

Gradient elution was performed using mobile phase A (MPA) consisting of 10 mM Na_2_HPO_4_, 10 mM sodium tetraborate decahydrate, in DI H_2_O, adjusted to pH 8.2 with HCl, filtered to 0.45 µm. Mobile phase B (MPB) consisted of 45:45:10 acetonitrile:methanol:DI H_2_O. Gradient used for elution was as follows: 0 min, 98% MPA, 2% MPB; 0.2 min, 98% MPA, 2% MPB; 6.67 min, 46% MPA, 54% MPB; 6.77 min, 0% MPA, 100% MPB; 7.3 min, 0% MPA, 100% MPB; 7.4 min, 98% MPA, 2% MPB; 8 min, 98% MPA, 2% MPB. Flow rate was 2.0 mL/min. The pump used was an Agilent Infinity Series G1311B Quat Pump. Precolumn derivatization was performed using an Agilent 1260 ALS (G1329B) with an injector program. Detection was performed using an Agilent 1260 Infinity II MWD (G7165A) at 338 nm with 10 nm bandwidth. Reference was 390 nm with 20 nm bandwidth. Recovery was tested during method development. Recoveries of cystine ranged from 87.2% to 101.5%, with an average of 92.1%.

### Methods to measure H_2_S using a microsensor

Aliquots of at least 1 mL were taken from cultures at desired times after inoculation. We used the Unisense H_2_S microsensor probe (https://unisense.com/products/h2s-microsensor/) following exactly the manufacturer’s method for making standards and calibrating. H_2_S concentrations were measured by suspending the H_2_S probe in the aliquot and leaving it in place until the measurement stabilized over 2 min. Because the measurement fluctuates over the course of these minutes, we excluded data gathered while the probe was stabilizing in the sample and averaged the value for each time point.

### Generation of metagenome-assembled genomes

Sequencing of the Lake Mendota time series for 2008–2012 was previously conducted at the Joint Genome Institute (26), containing 97 time points (and therefore 97 metagenomic datasets) (27). In summary, raw reads were quality filtered using Fastp (28) and individually assembled using metaSPAdes (29). Each metagenome was reciprocally mapped to each individual assembly using BBMap v38.07 (30), with 95% sequence identity cutoff. Differential coverage mapping to all samples was used to bin contigs into metagenome-assembled genomes (MAGs) using MetaBAT2 v.2.12.1 (31). Bins were quality assessed with CheckM v.1.1.2 (20), dereplicated with dRep v.2.4.2 (32), and classified with GTDB-tk v.0.3.2 (33) with default settings. This resulted in a total of 116 MAGs from Lake Mendota (Table S12 at https://doi.org/10.6084/m9.figshare.21711551), which are available for download at the Open Science Framework (https://osf.io/qkt9m/).

### Searching for cysteine genes and isolates presence in metagenomic time series and MAGs

Genes for cysteine degradation were identified using HMMsearch v3.1b2 (34). Hidden Markov models (HMMs) were downloaded from KOfam (35), accessed May 2020. The KEGG ontology (KO) numbers for the six cysteine degradation genes are: *metC* (K01760), *cysK* (K01738), *cysM* (K12339), *malY* (K14155), *tnaA* (K01667), and *dcyD* (K05396) (Table S4 at https://doi.org/10.6084/m9.figshare.21711491). The HMM files are those published by KOfam, with the modification of manual addition of the TC thresholds, curated by KEGG. HMM-based homology searches were conducted on the 97 Lake Mendota metagenome assemblies as described above.

## RESULTS

### Isolates capable of H_2_S production in oxic conditions

To answer the question of whether bacteria could produce H_2_S in the presence of oxygen, we grew freshwater isolates in pure culture originally recovered from the water column of temperate eutrophic Lake Mendota. We grew the isolates in moderately rich media (R2A, see Methods) under control and treatment conditions (cysteine addition) and tracked H_2_S production after 24 h (Fig. S1; Table S1 at https://doi.org/10.6084/m9.figshare.2171148 8). Using qualitative H_2_S measurements, we found that 18 isolates produced H_2_S and ammonia when grown in the presence of amended cysteine. We performed 16S rRNA gene sequencing on the 29 isolates that produced H_2_S, regardless of whether they produced ammonia or not when amended with cysteine. Isolates that produced both H_2_S and ammonia were identified as *S. rhizophila* (Betaproteobacteria), *S. maltophilia* (Betaproteobacteria), *Citrobacter gillenii* (Gammaproteobacteria), and *Chryseobacterium* sp. (Bacteroidota), whereas those producing H_2_S but not ammonia were identified as *Pseudomonas arsenicoxydans*, *P. mandelii*, *P. migulae*, *P. thivervalensis*, and *Mycobacterium flavescens*.

### Detailed microbiological, chemical, and genomic characterization in selected isolates

**Fig 2:**
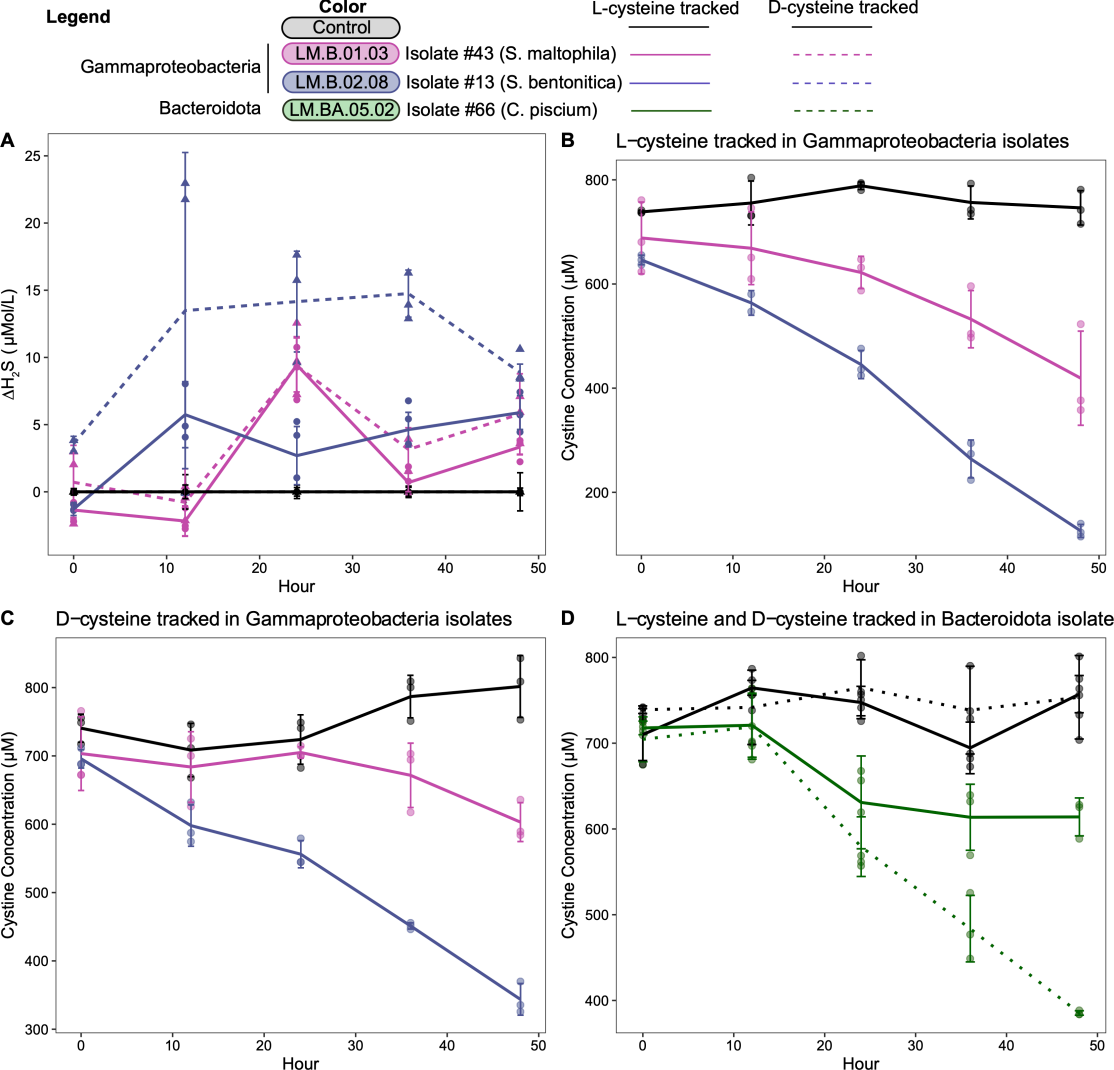
Quantitative characterization of cysteine utilization and hydrogen sulfide production in the three freshwater bacterial isolates using high-performance liquid chromatography (HPLC). (A) Higher amounts of H_2_S was produced by the isolates compared with negative controls (meaning no bacteria were grown in the culture) over the course of 50 h. (B–D) Identification of different forms of cysteine that can be degraded. L-cysteine decreased in all isolates compared with the control (B, D). D-cysteine also decreased over time in all samples except the negative control; however, the net amount decreased was less compared with L-cysteine. Cysteine concentrations were measured as cystine as described in the Methods section. Because Gammaproteobacteria isolates (#43 and #13) were assessed in a different experimental run than the Bacteroidota isolate (#66) but using the exact same instruments and methods, plots B, C, and D are separated by HPLC runs. Due to large sample volume, it was not possible to test all isolates and conditions in one HPLC run.

Next, we selected three isolates (#43, #13, and #66) representing distinct species based on 16S rRNA sequence taxonomy (97% identity), which produced H_2_S for further characterization. These detailed characterizations include OD_600_-based growth rates and paired quantitative measurements of cysteine and H_2_S concentrations in the spent medium. Cysteine addition resulted in concommital H_2_S production over time (Fig. 2A; Table S2 at https://doi.org/10.6084/m9.figshare.21711509). We also tested which forms of cysteine (L-cysteine or D-cysteine) the isolates used. The Gammaproteobacteria isolates used L- and D-cysteine at similar rates, but *C. piscium* used D-cysteine at a greater rate than L-cysteine (Fig. 2B to D; Table S3 at https://doi.org/10.6084/m9.figshare.21711497).

Next, we performed whole-genome sequencing using combined short-read and long-read sequencing on these three isolates. Isolate “13-LM-B-02-08” (referred to as #13) had a genome size of 4.18 Mbp, GC content of 66.8%, and was classified to the phylum Proteobacteria, class Gammaproteobacteria, order Xanthomonadales, family Xanthomonadaceae, genus *Stenotrophomonas*, and species *S. maltophilia*. Isolate #13 was fully circular and assembled in one scaffold. Isolate 43-LM-B-01-03 (referred to as #43) had a genome size of 4.3 Mbp, GC content of 66.5%, and was classified to the phylum Proteobacteria, class Gammaproteobacteria, and species *S. bentonitica*. Isolate #43 was assembled in two scaffolds. Finally, isolate LM_BA_5.2 (referred to as #66) had the smallest genome at 1.37 Mbp, GC content of 33.7%, and was classified to the phylum Bacteroidota, class Bacteroidia, order Flavobacteriales, family Weeksellaceae, genus *Chryseobacterium*, and species *C. piscium*. Isolate #66 was assembled in seven contigs. All three genomes were estimated to have ~100% genome completeness according to GTDB-tk.

We performed whole-genome sequencing because functional information such as gene content cannot be predicted reliably from 16S rRNA gene sequencing alone. The whole genome of isolate #43 was assembled into a single circular genome and taxonomically assigned to *S. maltophilia*. Unlike the 16S rRNA sequence that assigned it to *S. rhizophila*, the full genome was actually closer to *S. maltophilia*. The whole genome of isolate #13 could be assembled into two long contigs and was taxonomically assigned to *S. bentonitica*. The *Chryseobacter* genome was assembled into one circular genome and assigned to *C. piscium*. All three genomes were estimated to be 100% complete based on CheckM. Overall, the 16S rRNA gene amplicon sequencing performed prior agreed with full-genome sequencing assignment in some cases, and in others, the whole-genome sequencing assignment allowed finer taxonomic resolution (such as in the case of isolate #13).

**Fig 3:**
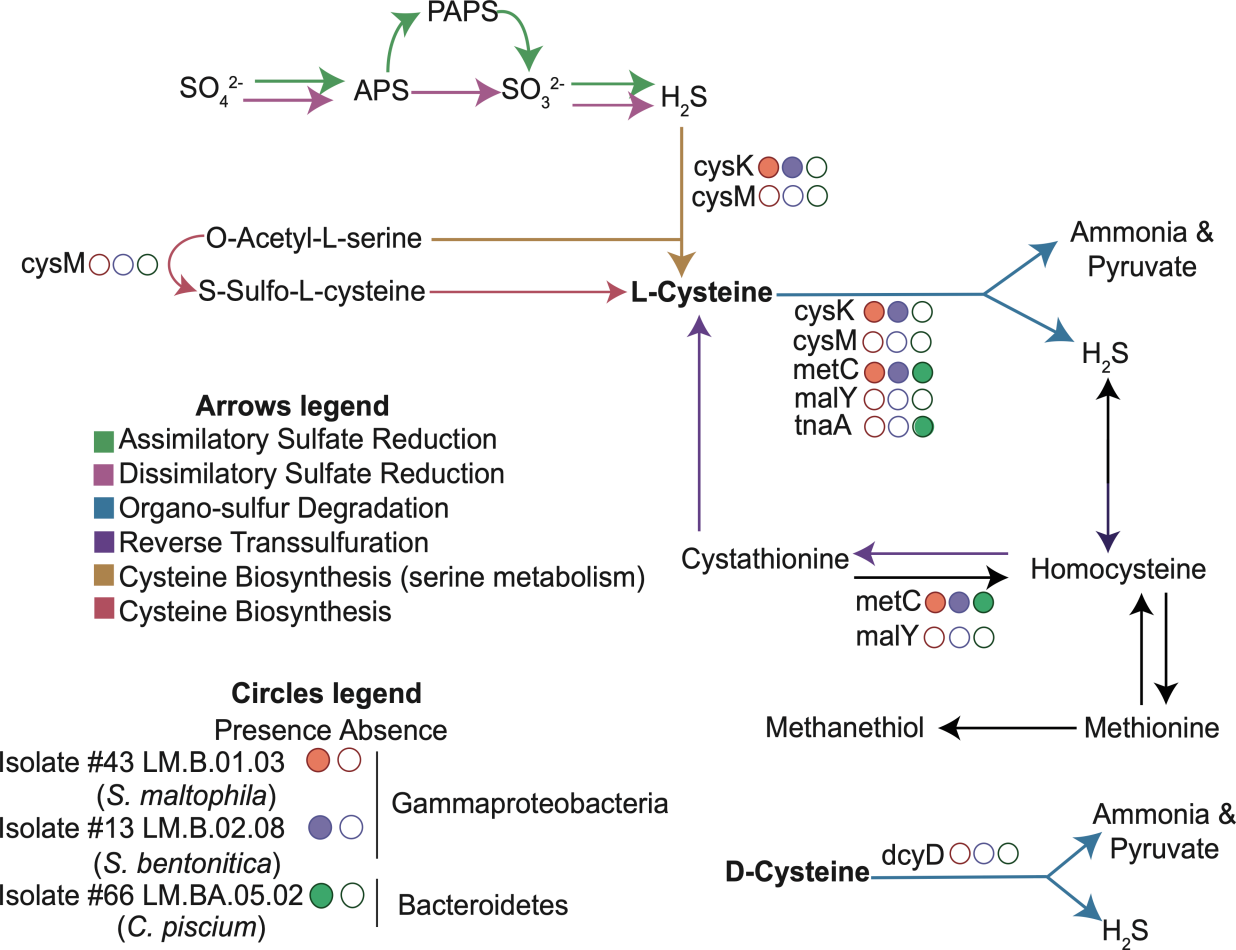
Genes involved in key pathways of sulfur and organosulfur metabolism in the three isolates. Several pathways for hydrogen sulfide and cysteine production exist in microorganisms. The presence/absence of key genes (*cysK*, *cysM*, *malY*, *metC*, *tnaA*, *sseA*, *aspB*, and *dcyD* along the blue arrows) in the three isolate genomes is shown by filled (present) circles and unfilled (not present) circles.

Overall, whole-genome sequencing provided more information about the isolates’ metabolic potential. The genomic content was then used to inform how or why H_2_S might be produced in oxic environments, as shown in the laboratory experiments. We used gene annotations of the three isolates to infer the presence of genes involved in cysteine metabolism, namely those involved in cysteine degradation to ammonia, pyruvate, and H_2_S: *metC*, *malY*, *tnaA*, *cysM*, *cysK* (which involve the use of L-cysteine as the substrate), and *dcyD* (which involves the use of D-cysteine as the substrate) (Table S4 at https://doi.org/10.6084/m9.figshare.21711491). However, we note that these genes may have other enzymatic activities, such as cysteine biosynthesis instead of degradation (Fig. 3; Table S5 at https://doi.org/10.6084/m9.figshare.21711494).

**Fig 4:**
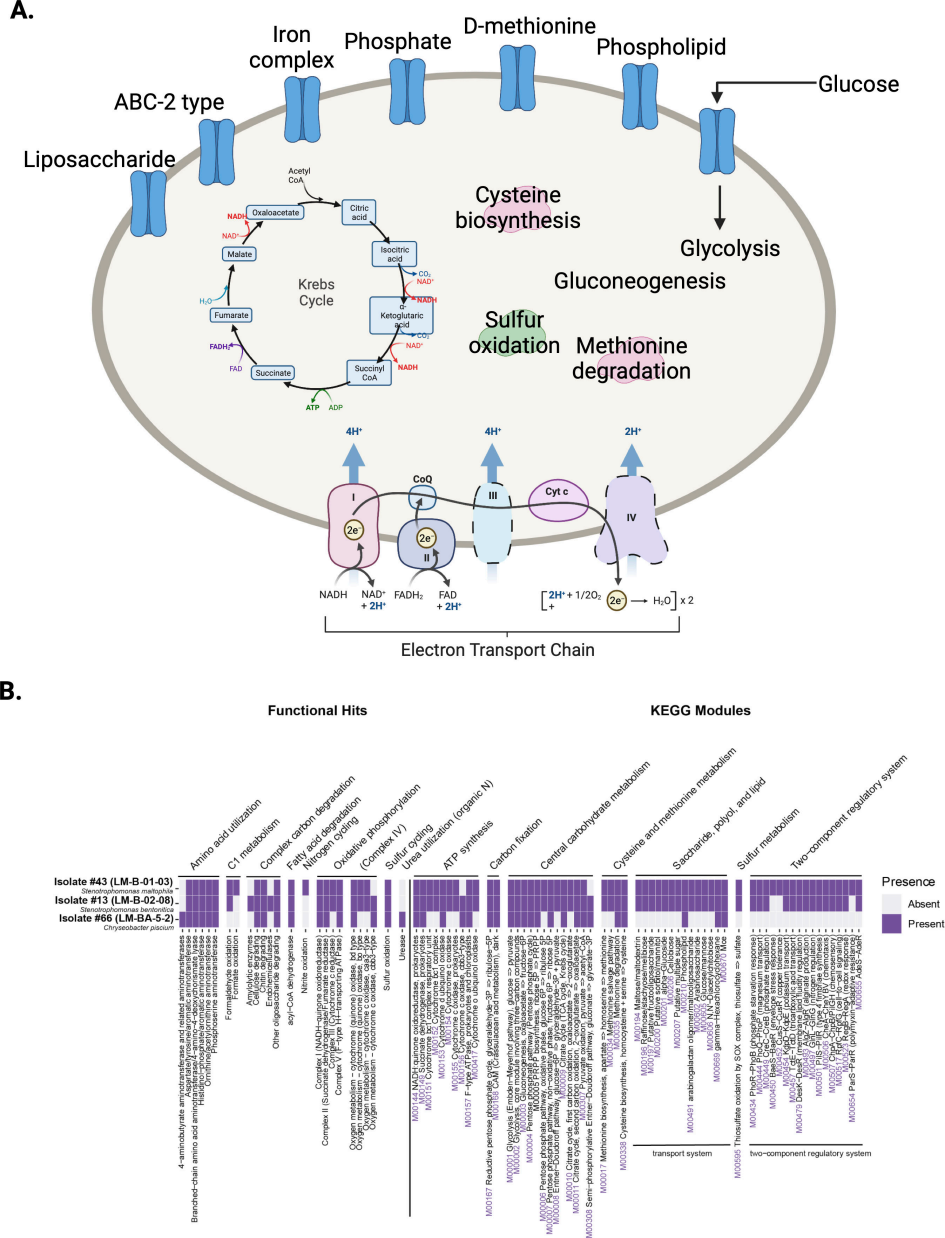
Common and distinguishing features of the three cysteine-degrading isolates. (A) Cellular map showing important metabolic pathways and transporters, which were common to all three isolates. A complete list is found in Table S6 (https://doi.org/10.6084/m9.figshare.21711500). The KEGG module identifiers are listed in purple whenever relevant. (B) Heat map showing selected metabolic functions and pathways in the three isolates. A complete list is found in Table S6.

Leveraging the full genomic content of the three isolates (Table S6 at https://doi.org/10.6084/m9.figshare.21711500), we proposed a joint cellular map based on identified metabolic functions and pathways in the genomes (Fig. 4A). All three isolates contained pathways associated with central carbon metabolism: including the TCA cycle, glycolysis, gluconeogenesis, the pentose phosphate pathway, and the glyoxylate cycle. They could all generate fatty acids using fatty acid biosynthesis and catabolize fatty acids through the beta-oxidation pathway. As expected, they had genes for cysteine metabolism, including *metC*, *malY*, *tnaA*, *cysM*, *cysK*, and *dcyD*, cysteine biosynthesis pathways from homocysteine and serine, as well as pathways for degradation of other amino acids, including methionine.

Despite these similarities, the three isolates also have distinguishing characteristics among them (Table S6 at https://doi.org/10.6084/m9.figshare.21711500; Fig. 4B). For example, while all isolates encoded genes for sulfur oxidation (sulfur dioxygenase), genes for thiosulfate oxidation were present in the two *Stenotrophomonas* isolates but not in *Chryseobacterium*. The *Chryseobacterium* isolate encoded for a urease, suggesting the use of organic nitrogen in the form of urea, but this was absent in the two *Stenotrophomonas* isolates. Finally, genes for sugar utilization were identified in the two *Stenotrophomonas* isolates but not in *Chryseobacterium*.

### Presence of cysteine-degrading organisms and genes in a 5-year metagenomic environmental time series

To put these laboratory results and lab-grown organisms into a natural environment context, we leveraged a previously published metagenomic time series collected from the oxygenated upper mixed layer of Lake Mendota spanning 2008–2012 (97 time points) (26) and reassembled and rebinned the data (see Methods) to search for the presence of cysteine degradation genes in the microbial communities.

First, we searched the time series to see if organisms in our study were also present in the time series. To do this, we linked the 16S rRNA gene sequences of the isolated organisms to the assembled metagenomes (i.e., contigs) from the time series. We found that while the 16S rRNA sequences were also present in the time series (Table S7 at https://doi.org/10.6084/m9.figshare.21711530, S8 at https://doi.org/10.6084/m9.figshare.21711536, and S9 at https://doi.org/10.6084/m9.figshare.217115 48), and broadly distributed over time, these scaffolds were not part of binned genomes. Therefore, little information about these 231 isolates would be gathered from metagenomic data only. As such, the full-genome sequencing we performed was particularly helpful in understanding the full genomic structure of the H_2_S-producing organisms.

**Fig 5:**
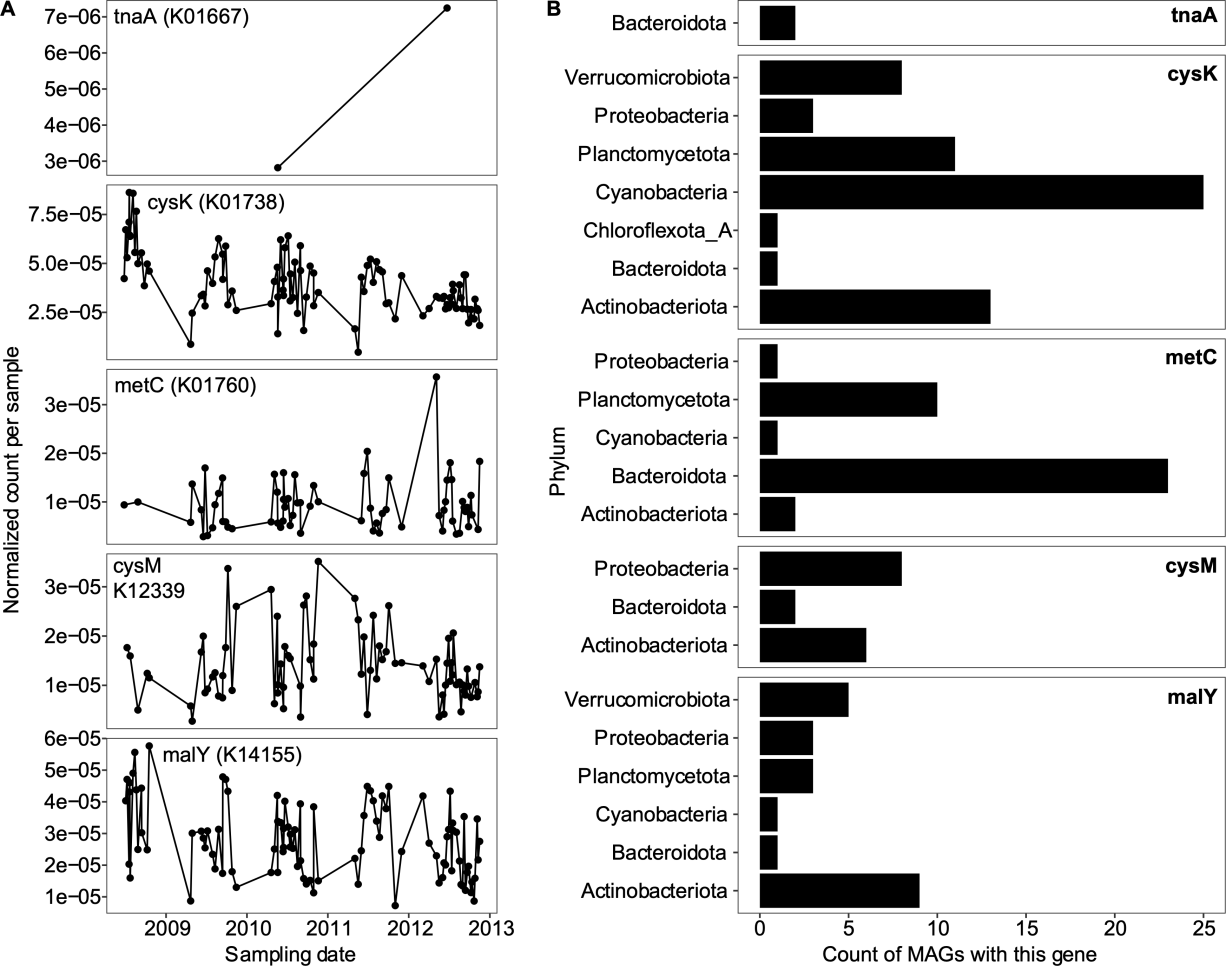
Distribution of cysteine desulfurylation genes across time and taxa. (A) Counts of cysteine desulfurylation genes (five genes) in a 5-year time series of Lake Mendota, normalized by total number of annotated genes per metagenome sample. We also searched for *dcyD* but did not identify it in any sample. (B) Taxonomy of the metagenome-assembled genomes in which those genes were found.

Second, we searched for the six genes associated with cysteine degradation and H_2_S production (Table S5 at https://doi.org/10.6084/m9.figshare.21711494 ) (in binned and unbinned contigs) using KEGG HMMs with HMMsearch. In total, we searched over 22 million amino acid sequences and identified 1,882 hits to the five marker genes found in the isolate metagenomes; *dcyD* was not found (Fig. 5; Table S10 at https://doi.org/10.6084/m9.figshare.21711527). *cysK* and *malY* were the genes with the most corresponding matches at any time point, followed by *metC* and *cysM*. Only two scaffolds contained *tnaA*. Overall, after normalizing for metagenome read size per sample, there was no obvious temporal trend of the genes, although genes were found throughout the 5-year time series.

Among these cysteine-degrading gene sequences, several were identified in binned MAGs (Fig. 5B; Table S11 at https://doi.org/10.6084/m9.figshare.2171154 5), which allowed for the assignment of taxonomy. Overall, we identified 139 genes to be distributed in genomes of organisms from Actinobacteria, Bacteroidota, Chloroflexota, Cyanobacteria, Planctomycetes, Proteobacteria, and Verrucomicrobia, representing common freshwater lineages (36). The *tnaA* gene was only present in Bacteroidota, but other genes were more broadly distributed among taxonomic groups.

## DISCUSSION

### The fate of H_2_S and ammonia, two products of cysteine degradation

H_2_S blocks the binding of oxygen during aerobic respiration, making it toxic to the cell (37). Therefore, we hypothesize that most microbes aerobically degrading cysteine will excrete H_2_S from the cell, as was seen with the 29 isolates from this study producing H_2_S when grown in media containing a cysteine source. Additionally, the three isolates that underwent further characterization were quantitatively shown to deplete cysteine levels when producing H_2_S (Fig. 2). While it is possible that organisms may use H_2_S internally as a sulfur source, we did not identify any genes encoding sulfide quinone oxidoreductases, flavocytochrome c sulfide dehydrogenases, or other genes for the oxidation or transformation of H_2_S. H_2_S can also be used as a protective compound against antibiotics in aerobic environments by some bacteria; however, due to the stress H_2_S accumulation has on the cell, this defense mechanism is only used in extreme situations (37).

The H_2_S-producing isolates fell into two groups when grown in media with cysteine: ammonia producing and ammonia consuming. Because ammonia is a common metabolic precursor, the production or consumption of ammonia alone does not indicate whether the cell is undergoing the cysteine degradation pathway. This is likely why we see variation in whether the 29 isolates produced or consumed ammonia despite all producing H_2_S (Fig. S1).

### Genomic structure of the H_2_S-producing isolates

Overall, the three isolates selected for whole-genome sequencing revealed genes for cysteine degradation into H_2_S. Based on laboratory studies, they were able to produce H_2_S in the presence of oxygen. The isolates were obligate aerobes, presenting interesting questions about these organisms’ life history.

*S. maltophilia*, *S. bentonitica*, and *C. piscium* have been shown to be present in natural environments. *S. maltophilia* is a cosmopolitan bacterium in nature and found in a range of natural environments, particularly in association with plants (38). *S. bentonitica* was originally characterized in bentonite formations, was predicted to have high tolerance to heavy metals (39), and has been observed in arctic seawater (40). *C. piscium* was isolated from a fish in the arctic ocean (41), but its ecological significance in the oceans remains unknown. This previously described *C. piscium* strain LMG 23089 was not reported to produce H_2_S, yet our genetic and physiological analyses suggest that it has the enzymatic machinery to degrade cysteine.

One possible explanation for this discrepancy is that LMG 23089 was previously grown on sulfur reduction, indole production, and motility (SIM) medium to test H_2_S. The SIM medium is used for physiological study of SIM. The SIM medium offers visual representation of H_2_S production via the reduction of thiosulfate; it reacts with iron salts in the media, and media changes to darker black color. This method of assessing H_2_S is of lower resolution than the modern H_2_S probes that measure µM concentrations. As a side test on isolate #66, H_2_S was not produced when thiosulfate was provided, but H_2_S was produced when cysteine was provided.

One particular finding of this study was that none of the six genes searched for cysteine degradation into H_2_S and ammonia was common to *all* three isolates, despite all three isolates showing the same cysteine decrease, ammonia increase, and H_2_S increase over time. This could be explained by alternative, perhaps less straightforward pathways for H_2_S production. One pathway is led by a gene named cystathionine gamma-lyase (“CTH” or “CSE”). In some bacteria and mammals, this enzyme is involved in H_2_S production (42). An HMM search for this enzyme showed that it was present in isolates #13, #43, and #66. While it was not initially included in the initial methods and study, this could hint to another commonality among oxic H_2_S-producing organisms.

### Challenges associated with measuring oxic H_2_S production from organosulfur in the environment

Extrapolating these laboratory results to widespread distribution of organosulfur degradation in the natural environment necessitates several steps, namely because of the major knowledge gaps that exist concerning the sulfur cycle in freshwater lakes and because bridging the gap between cultivation-based, omics-based (43), and field-based experiments is needed. Foremost, the identity, distribution, and availability of organosulfur compounds broadly across lakes globally are currently mostly unknown. Cysteine is notoriously difficult to measure, and many previous studies characterizing the amino acid composition of the water column only measure the sulfur-containing organosulfur compound taurine (44, 45). One of the difficulties in studying the fate of cysteine in oxic environments is that it can be abiotically and spontaneously oxidized into cystine (24), which *E. coli* has been shown to uptake (25). In a study of *E. coli* K-12 that lacked a cysteine transporter, cysteine could enter the cell through transporters dedicated to other amino acids, when no amino acid alternatives other than cysteine were present in the medium (14).

Organic sulfur in the form of cysteine is an important organosulfur amino acid and is important in protein folding and function (46). As such, there is a difference in the fates of cysteine when it exists bound in cell walls versus when cysteine is free in the water column and available for degradation by bacteria. While cysteine has been shown to contribute to the carbon pool and carbon flow in lakes (6), more quantitative field measurements are necessary to support whether cysteine also serves as a sulfur pool. Yet, other forms of organosulfur have important significance in aquatic environments. In marine environments, for example, dimethylsulfoxonium propionate is a critical component of the marine organosulfur cycle (47).

Additionally, current differences between computational gene similarity searches versus *in vivo* enzymatic functions are challenging to assess for the genes responsible for the cysteine degradation into pyruvate, ammonia, and H_2_S. One reason is that the enzymatic activity of the gene has mostly been described in model organisms such as *E. coli*, and it has been shown that gene expression can be induced by genetic factors or environmental factors such as metals (48). At least six genes have been proposed to have this enzymatic activity, yet each gene may serve different functions *in situ*, and it is difficult to assert directionality of enzymatic function based on metagenomic or genomic analyses only. To this end, the isolated bacterial strains from this study, which are non-model organisms and originate from the natural freshwater lake environment, may be used for further detailed biochemical, physiological, and microbiological studies. Further characterization of these bacterial isolates using gene knockout, gene induction, or heterologous gene expression studies may inform the functional activity of these genes in nature.

### Implications of oxic H_2_S production by microbes in freshwaters

This study demonstrates the potential for H_2_S production by microbes in lake ecosystems to occur in the presence of oxygen, using genomic and physiological evidence from pure culture bacterial isolates and screening of long-term metagenomic time series. By combining lake-to-laboratory experiments, we show that multiple bacterial strains spanning Gammaproteobacteria, Betaproteobacteria, Actinobacteria, and Bacteroidota are all able to produce H_2_S under oxic conditions and at temperatures that would be ecologically relevant for surface lake water during the summer. Surface water temperatures in Lake Mendota can reach up to 27°C, and the top few meters of water surface are saturated in oxygen. Worldwide, maximum lake surface temperature can range between 23°C and 31°C (49).

Unlike dissimilatory sulfate reduction, bacteria use cysteine to generate ammonia, pyruvate, and H_2_S and are not dependent on sulfate as an initial reaction substrate. Increased sulfate concentrations are shown to lead to higher sulfate reduction rates in shallow eutrophic freshwater, the sulfur originating from algal decay, for example (50). While Lake Mendota is a low-iron and high-sulfate lake (51), not all lakes have elevated sulfate levels, and therefore, H_2_S production might previously not have been thought of as relevant to study. However, sulfur-containing amino acids can have many origins. In lakes, concentrations of amino acids (free dissolved and combined) often reflect the input and outputs of the lake (52, 53). For example, amino acids contributed a detectable amount to the nitrogen cycle, and bacterial utilization of amino acids contributes to nitrogen pool and cycling in natural ecosystems (53), although cysteine amino acids were not measured in that study.

**Fig 6:**
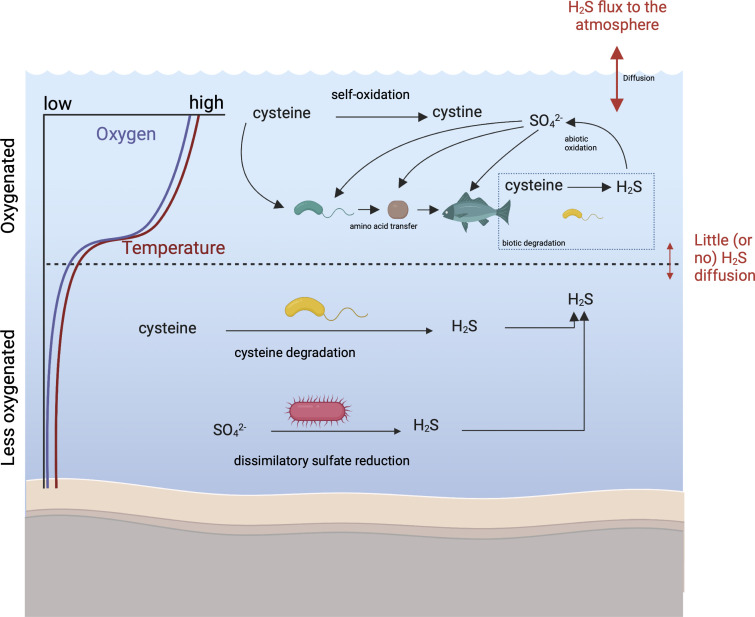
Conceptual figure showing the potential relevance of cysteine degradation in a freshwater lake environment, with respect to oxygen availability and seasonality. Oxygenated seasons and part of the lake water columns are shown with an asterisk. Significant research gaps include cysteine concentrations in the natural environment over time, hypothesized H_2_S fluxes across different layers in the lake water column, and contribution of different H_2_S sources in the hypolimnion. In all seasons, portions of the water column can be oxygenated.

Freshwater lakes that are dimictic can become stratified in temperature and oxygen during the summer, and oxygen concentrations vary throughout the year. In the fall and spring, oxygen is abundant, and cysteine degradation into H_2_S could be a relevant process for the sulfur pool, and H_2_S fluxes to the atmosphere could be significant since wind is prevalent. Under ice during the winter, where oxygen is plentiful, H_2_S could be produced but could be consumed or oxidized. On the other hand, gases would be trapped under ice. During summer, the anoxic hypolimnion and sediments are known H_2_S sources due to dissimilatory sulfate reduction, but density gradients would prevent H_2_S from reaching the atmosphere. However, the oxygenated mixed epilimnion could be an H_2_S source through organosulfur degradation. If we consider the importance of oxic H_2_S production, which could occur year-round, the H_2_S pool and the scope of sulfur transformations may be greater than anticipated, if we focus solely on the anoxic hypolimnion (Fig. 6). Future work aiming to understand the broader distribution of sulfur-containing amino acids and other organosulfur compounds in freshwaters, their fates and transformations, as well as their contribution to H_2_S production, will inform global sulfur biogeochemical cycling.

## Data Availability

The 16S rRNA sequences for the 29 H2S-producing isolates, and the whole-genome sequences (nucleotides and amino acids) for isolates #13, #43, and #66 are available on OSF at the following DOI: https://doi.org/10.17605/OSF.IO/G25EQ. The isolates genomes are also deposited on NCBI in the Bioprojects: PRJNA776273, PRJNA776272, and PRJNA839079. The 97 metagenomes were previously published in reference 45 and are available through JGI’s IMG/M and Genome Portal.

## SUPPLEMENTAL MATERIAL

Fig. S1 msystems.00201-23-s0001.pdf Qualitative accumulation of hydrogen sulfide among microbial isolates enriched from a freshwater lake water column. Filled circles represent isolates grown with cysteine, and open circles represent isolates grown without cysteine. The vertical line represents values corrected for the control (natural ammonia production/consumption in the negative control). All points to the right of the vertical lines indicate an accumulation of ammonia, and all points to the left of the vertical lines refer to those that consumed ammonia after 24 h. The isolates #43, #13, and #66 (bolded) were selected for further analysis.

ASM does not own the copyrights to Supplemental Material that may be linked to, or accessed through, an article. The authors have granted ASM a non-exclusive, world-wide license to publish the Supplemental Material files. Please contact the corresponding author directly for reuse.
