# Supplementary figures and images for: Physiological and genomic evidence of cysteine degradation and aerobic hydrogen sulfide production in freshwater bacteria
Source: mSystems. 2023 Jun 7;8(3):e00201-23. doi: 10.1128/msystems.00201-23 (PMC10308919; doi:10.1128/msystems.00201-23)

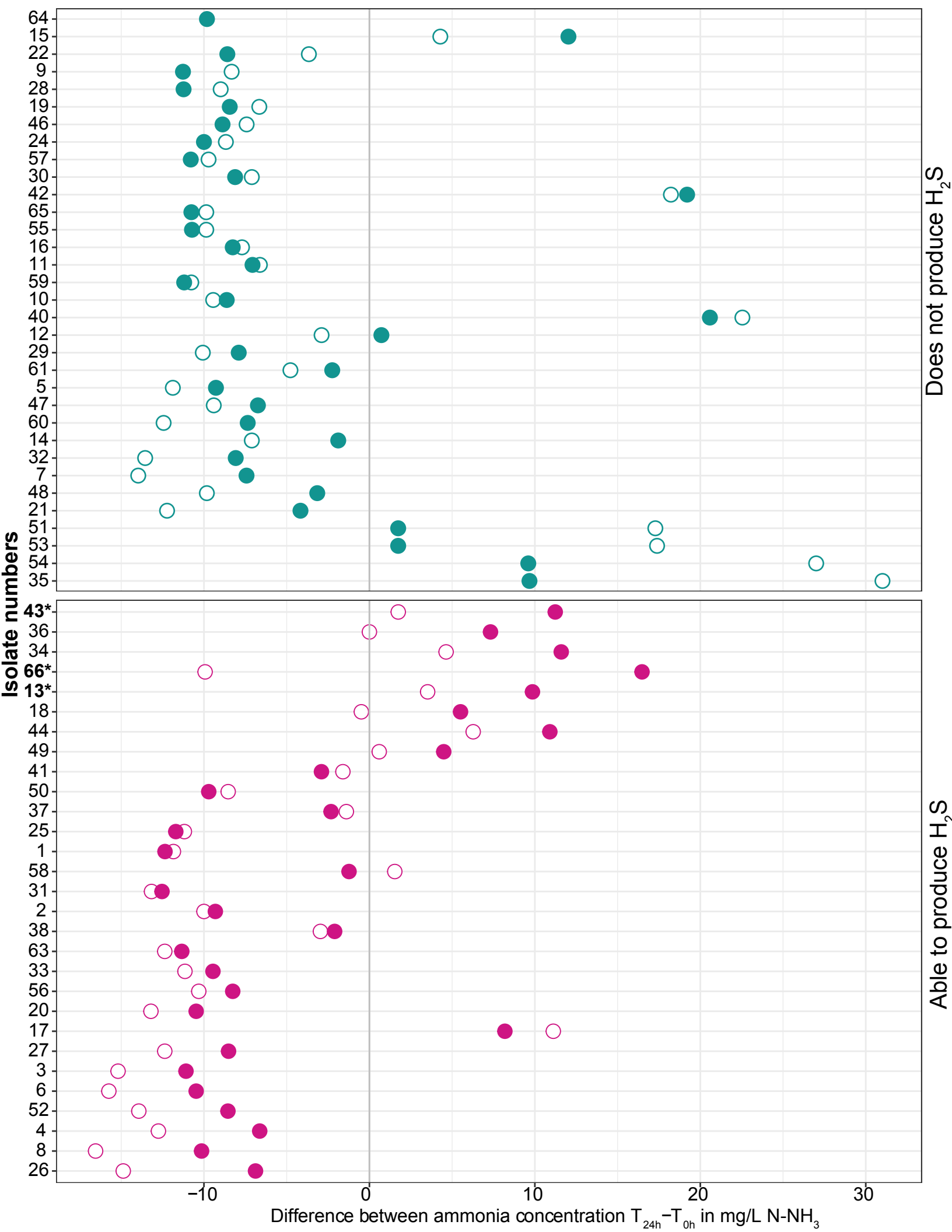

Supplement: Fig. S1 — Qualitative accumulation of hydrogen sulfide among microbial isolates enriched from a freshwater lake water column. Filled circles represent isolates grown with cysteine, and open circles represent isolates grown without cysteine. The vertical line represents values corrected for the control (natural ammonia production/consumption in the negative control). All points to the right of the vertical lines indicate an accumulation of ammonia, and all points to the left of the vertical lines refer to those that consumed ammonia after 24 h. The isolates #43, #13, and #66 (bolded) were selected for further analysis. [file msystems.00201-23-s0001.pdf]
